# Supplementary material for: Ancient DNA reveals the lost domestication history of South American camelids in Northern Chile and across the Andes
Source: eLife. 2021 Mar 16;10:e63390. doi: 10.7554/eLife.63390 (PMC8032396; doi:10.7554/eLife.63390)
Supplement: Transparent reporting form [file elife-63390-transrepform.pdf]

eLIFE
1st Floor
24 Hills Road
Cambridge CB2 1JP, UK
P 01223 855340
W elifesciences.org
T @elife

eLife's transparent reporting form

We encourage authors to provide detailed information *within their submission* to facilitate the interpretation and replication of experiments. Authors can upload supporting documentation to indicate the use of appropriate reporting guidelines for health-related research (see EQUATOR Network), life science research (see the BioSharing Information Resource), or the ARRIVE guidelines for reporting work involving animal research. Where applicable, authors should refer to any relevant reporting standards documents in this form.

If you have any questions, please consult our Journal Policies and/or contact us: editorial@elifesciences.org.

Sample-size estimation

- You should state whether an appropriate sample size was computed when the study was being designed
- You should state the statistical method of sample size computation and any required assumptions
- If no explicit power analysis was used, you should describe how you decided what sample (replicate) size (number) to use

Please outline where this information can be found within the submission (e.g., sections or figure legends), or explain why this information doesn't apply to your submission:

Sample size estimation can be found:
- Main manuscript, section Materials and methods:
  Pag. 22, *Ancient DNA extraction for ancient samples;*
  Pag. 24, *data set of new modern DNA samples*.
- Supplementary material: Tables S1, S2 and S3.

Replicates

- You should report how often each experiment was performed
- You should include a definition of biological versus technical replication
- The data obtained should be provided and sufficient information should be provided to indicate the number of independent biological and/or technical replicates
- If you encountered any outliers, you should describe how these were handled
- Criteria for exclusion/inclusion of data should be clearly stated
- High-throughput sequence data should be uploaded before submission, with a private link for reviewers provided (these are available from both GEO and ArrayExpress)

Please outline where this information can be found within the submission (e.g., sections or figure legends), or explain why this information doesn't apply to your submission:

- Main manuscript, section Materials and methods:
  -Morphometric analysis: pag.21
  -Ancient DNA bioinformatics processing pag. 23
- Suplemmentary material: Table S5.

Data deposition: Thesequences reported in this paper havebeen deposited in theGenBank database

eLife Sciences Publications, Ltd is a limited liability non-profit non-stock corporation incorporated in the State of Delaware, USA, with company number 5030732, and is registered in the UK with company number FC030576 and branch number BR015634 at the address 1st Floor, 24 Hills Road, Cambridge CB2 1JP | August 2014

Statistical reporting

- Statistical analysis methods should be described and justified
- Raw data should be presented in figures whenever informative to do so (typically when N per group is less than 10)
- For each experiment, you should identify the statistical tests used, exact values of N, definitions of center, methods of multiple test correction, and dispersion and precision measures (e.g., mean, median, SD, SEM, confidence intervals; and, for the major substantive results, a measure of effect size (e.g., Pearson's r, Cohen's d)
- Report exact p-values wherever possible alongside the summary statistics and 95% confidence intervals. These should be reported for all key questions and not only when the p-value is less than 0.05.

Please outline where this information can be found within the submission (e.g., sections or figure legends), or explain why this information doesn't apply to your submission:

Main manuscript- section Materials and methods:

-*Ancient DNA bioinformatic processing. Pag23*
- *Phylogenetic and Demographic Analysis using mitogenome data*. Pag24

(For large datasets, or papers with a very large number of statistical tests, you may upload a single table file with tests, Ns, etc., with reference to sections in the manuscript.)

Group allocation

- Indicate how samples were allocated into experimental groups (in the case of clinical studies, please specify allocation to treatment method); if randomization was used, please also state if restricted randomization was applied
- Indicate if masking was used during group allocation, data collection and/or data analysis

Please outline where this information can be found within the submission (e.g., sections or figure legends), or explain why this information doesn't apply to your submission:

Data collection:
Figure 1.Pag3. Collection of modern samples.
Material and methods: Archaeological samples. Pag20.

Additional data files ("source data")

- We encourage you to upload relevant additional data files, such as numerical data that are represented as a graph in a figure, or as a summary table
- Where provided, these should be in the most useful format, and they can be uploaded as "Source data" files linked to a main figure or table
- Include model definition files including the full list of parameters used
- Include code used for data analysis (e.g., R, MatLab)
- Avoid stating that data files are "available upon request"

Please indicate the figures or tables for which source data files have been provided:

Supplementary tables:
Table S1: List of selected camelid phalanx and astragalus bones from Tulán-54 and Tulán-85 for DNA extraction.
Table S2: List of partial mitochondrial genomes sequences of modern camelids included in the Haplotype Network analysis.
Table S3: List of whole mitochondrial genomes sequences of modern camelids included in the Bayesian camelid´s phylogeny.
Table S4.1: Measurements of camelid bones.
TableS4.2: Measurements of camelid astragalus selected from Tulán-54 and Tulán-85.
Table S4.3.: Measurements (mean) of camelid first phalanx (anterior) and astragalus from modern samples (Cartajena 2003; L´Hereux, 2010)
Table S5: Information data about specimens used in th study and sequencing results.
Table S6: Output from PartitionFinder for analysis in MrBayes.
Table S7: Variation at 76 polimorphic sites among 158 haplotypes of South American camelids. Hypervariable I Domain sequences (Control Region, mtDNA).
Table S8: Genetic diversity indices: Number of haplotypes, haplotype diversity and nucleotide diversity, from all the mitogenomes clasified according to species (modern samples) or morphometric size (ancient samples).
Table_S9: List of haplotypes shared by modern and ancient samples in the Temporal network. Highlight in yellow is the ancient samples included in each shared haplotype.

Main Manuscript:
Figure 1. A. Map of the current geographic distribution of *Lama guanicoe* subspecies (based on WCS, 2013), white and black squares correspond to sampling locations of modern mitogenomes and CR hypervariable I domain respectively. B. Map of the current geographic distribution of *Vicugna vicugna* subspecies (based on González et al. 2020), white and black dots correspond to sampling locations of modern mitogenomes and CR hypervariable I domain respectively. C. Star shows the Tulán site in Atacama Desert in Chile, where the ancient samples (3,500 - 2,400 years before the present) were obtained.
Figure 2. Correlation graphics of anterior phalanx and astragalus measurements.
Figure 3. Mitogenomic phylogenetic tree.
Figure 4. Bayesian Skyline Plot (BSP) derived from the analysis of the ancient and modern camelid data.
Figure 5. Minimum spanning network of ancient and modern camelid partial control region sequences.
Figure 6. Temporal statistical parsimony haplotype networks for *Lama guanicoe, Lama glama, Vicugna vicugna and Vicugna pacos* with ancient samples.
Figure 7. Expected distributions of summaries of genetic diversity conditional on the aDNA sample size.

eLife Sciences Publications, Ltd is a limited liability non-profit non-stock corporation incorporated in the State of Delaware, USA, with company number 5030732, and is registered in the UK with company number FC030576 and branch number BR015634 at the address 1st Floor, 24 Hills Road, Cambridge CB2 1JP | August 2014
